# Evaluation of a Novel Reporter Virus Neutralization Test for Serological Diagnosis of Zika and Dengue Virus Infection

**DOI:** 10.1128/JCM.00975-17

**Published:** 2017-09-25

**Authors:** Chao Shan, Daniel A. Ortiz, Yujiao Yang, Susan J. Wong, Laura D. Kramer, Pei-Yong Shi, Michael J. Loeffelholz, Ping Ren

**Affiliations:** a Department of Biochemistry & Molecular Biology, University of Texas Medical Branch, Galveston, Texas, USA; b Department of Pathology, University of Texas Medical Branch, Galveston, Texas, USA; c College of Animal Science and Technology, Southwest University, Chongqing, China; d Wadsworth Center, New York State Department of Health, Albany, New York, USA; Boston Children's Hospital

**Keywords:** Zika virus, dengue virus, neutralization assay, sensitivity, specificity

## Abstract

Currently, the laboratory diagnosis of Zika virus (ZIKV) infection is primarily through the detection of ZIKV RNA or antibodies against ZIKV proteins. The detection of viral RNA is highly sensitive and specific, but periods of viremia and viruria are brief, limiting the utility of ZIKV RNA assays. Instead, most ZIKV infections are diagnosed serologically, using an IgM antibody capture enzyme-linked immunosorbent assay (MAC-ELISA) for screening, followed by a confirmatory plaque reduction neutralization test (PRNT). Typical turnaround times vary, due to assay incubation periods and a lack of clinical laboratories performing these tests. Recently, a novel luciferase-ZIKV- and -dengue virus (DENV)-based serological assay, which considerably improves the turnaround times and throughput for ZIKV diagnosis, was described. Using the traditional PRNT as a reference method, we evaluated the performance characteristics of the reporter virus neutralization test (RVNT) with 258 clinical serum specimens. The ZIKV RVNT produced primary ZIKV screening and secondary confirmation results in 4 days, with 100% reproducibility. As a screening assay, the ZIKV RVNT displayed excellent diagnostic accuracy, sensitivity, and specificity of 98.2%, 100%, and 98.1%, respectively. As a confirmatory assay, the ZIKV RVNT titers displayed 93.1% agreement with the traditional ZIKV PRNT titers. Overall, the RVNT accurately and reliably detects neutralizing antibodies in patient serum specimens, with improved turnaround times, and can be used for the serological detection of ZIKV infections. Due to the homogeneous 96-well format, the RVNT has also significantly improved the assay throughput to allow testing of a large number of specimens in a single run.

## INTRODUCTION

Zika virus (ZIKV) is an enveloped, single-stranded RNA virus belonging to the genus Flavivirus. Similar to other clinically relevant flaviviruses (e.g., dengue virus [DENV], West Nile virus [WNV], Japanese encephalitis virus, and yellow fever virus), ZIKV is transmitted to humans primarily via the bites of infected mosquitos. It can also be transmitted through sexual, mother-to-fetus, and blood transfusion routes (1). The Aedes mosquito vector that transmits ZIKV is also capable of transmitting DENV and chikungunya virus. The regions in which they are endemic and the symptoms that are associated with these three mosquito-borne viruses overlap considerably, making definitive diagnosis challenging for clinicians (2–4). Diagnostic tests can aid in the identification of the etiological agent, which can significantly affect patient care and management.

Diagnostic testing for ZIKV infection can be accomplished using both molecular and serological methods. Molecular methods are used in early stages of infection to detect ZIKV RNA from a variety of sources, including blood, serum, plasma, saliva, semen, and cerebrospinal fluid (CSF). The detection of ZIKV RNA confirms ZIKV infection, but detectable levels of ZIKV RNA are transient in blood (≤14 days after symptom onset or exposure) and differ from person to person (5–7). Alternatively, ZIKV infections of ≥14 days can be identified through the serological detection of anti-ZIKV antibodies, which appear when RNA levels decline and become undetectable.

The current algorithm for serological ZIKV testing involves an antibody screen followed by a confirmatory test. Initial ZIKV antibody screens are conducted using an IgM antibody capture enzyme-linked immunosorbent assay (MAC-ELISA), with serum specimens from patients meeting the Centers for Disease Control and Prevention (CDC) clinical and epidemiological testing criteria (8). A nonreactive MAC-ELISA screen can rule out a recent ZIKV infection, while equivocal or presumptively positive specimens require confirmatory testing due to cross-reactivity with closely related flaviviruses, particularly DENV. When confirmatory testing is warranted, a plaque reduction neutralization test (PRNT) that quantifies neutralizing antibody titers to ZIKV and DENV in patient serum is performed. Based on the levels of ZIKV and DENV neutralization, a MAC-ELISA result can be either confirmed or considered a false-positive result. The PRNT can take 1 week or longer to perform, due to long incubation periods, low throughput, and a lack of clinical laboratories performing the assay. Consequently, patient results can be delayed, negatively affecting patient care.

Recently, a reporter virus neutralization test (RVNT), which considerably improved the turnaround time and assay throughput for ZIKV results, was described (9). The principle of the RVNT is similar to that of the PRNT, in that both assays quantify neutralizing antibody titers. However, the RVNT uses luciferase-tagged ZIKV and DENV, which allows quantification of neutralizing antibodies in 24 h, instead of the typical 7-day period required for plaques to become visible with the PRNT method. Also, the RVNT is performed in a 96-well-plate format, which enables high-throughput testing. The rapid turnaround time and high-throughput testing make the RVNT suitable for ZIKV screening and confirmatory testing. In this study, we evaluated the performance characteristics and utility of the RVNT as a ZIKV screen and confirmatory test, by analyzing clinical serum specimens from low- and high-risk patients.

## RESULTS

### Rationale for primary screening and secondary confirmation for RVNT diagnosis.

The goal of this study was to validate the rapid, high-throughput RVNT to potentially replace the labor-intensive, low-throughput PRNT. To reduce costs and to maintain a good turnaround time for testing, we adopted a two-step approach for RVNT diagnosis, by performing a primary screen followed by a secondary confirmation. The primary screen uses the RVNT to test each patient specimen at a single dilution of 1:10. For primary-screen-positive specimens, the secondary confirmatory test is performed to determine the RVNT 90% neutralizing titer (RVNT_90_). Both the primary screen and the secondary confirmation could be completed within 2 days. Therefore, diagnostic results for negative specimens could be released within 2 days, whereas diagnostic results for positive specimens could be released within 4 days.

### ZIKV screening.

All samples were first subjected to the primary screen using the RVNT, at a 1:10 dilution. Two groups of clinical serum specimens were included, i.e., one group of specimens from low-risk patients and one group of specimens from high-risk patients. The low-risk cohort consisted of 125 serum samples from individuals who had not traveled to ZIKV-affected areas after 2013, which were submitted for non-ZIKV-related testing, while the high-risk cohort contained 102 samples from patients who met the CDC testing criteria (which include symptoms, pregnancy, and exposure risk). Among the 125 low-risk patient specimens, the RVNT displayed excellent diagnostic accuracy for ZIKV and DENV (99.2% and 94.4%, respectively), compared to the traditional PRNT method (Table 1). Among the high-risk patient specimens, the diagnostic accuracy values for ZIKV and DENV were 97.1% and 89.2%, respectively (Table 1). Collectively, 227 patient samples were screened, and the results were compared to results obtained with the PRNT reference method. The ZIKV RVNT displayed 98.2% (95% confidence interval [CI], 95.4 to 99.5%) diagnostic accuracy, with 100% (95% CI, 74.9 to 100%) sensitivity and 98.1% (95% CI, 95.1 to 99.4%) specificity, while the DENV RVNT displayed 92.1% (95% CI, 87.8 to 95.0%) diagnostic accuracy, with 100% (95% CI, 83.1 to 100%) sensitivity and 91.2% (95% CI, 86.4 to 94.4%) specificity (Table 2).

**TABLE 1:** Comparison of RVNT and PRNT results for clinical specimens

Virus tested and RVNT result	No. of specimens with PRNT result of:		RVNT sensitivity (95% CI) (%)	RVNT specificity (95% CI) (%)	RVNT diagnostic accuracy (95% CI) (%)
	Positive (titer ≥ 10)	Negative (titer < 10)			
Low-risk cohort (*n* = 125)					
ZIKV			NA^*a*^	99.2 (95.2–100)	99.2 (95.2–100)
Positive (titer ≥ 10)	0	1			
Negative (titer < 10)	0	124			
DENV			100 (38.3–100)	94.3 (88.4–97.4)	94.4 (88.7–97.5)
Positive (titer ≥ 10)	3	7			
Negative (titer < 10)	0	115			
High-risk cohort (*n* = 102)					
ZIKV			100 (74.9–100)	96.6 (90.0–99.3)	97.1 (91.3–99.4)
Positive (titer ≥ 10)	14	3			
Negative (titer < 10)	0	85			
DENV			100 (81.0–100)	86.6 (77.4–92.5)	89.2 (81.6–94.0)
Positive (titer ≥ 10)	20	11			
Negative (titer < 10)	0	71			

a NA, not applicable.

**TABLE 2:** Overall performance characteristics of the RVNT as a screening method (*n* = 227)

Virus tested and RVNT result	No. of specimens with PRNT result of:		RVNT sensitivity (95% CI) (%)	RVNT specificity (95% CI) (%)	RVNT diagnostic accuracy (95% CI) (%)
	Positive (titer ≥ 10)	Negative (titer < 10)			
ZIKV			100 (74.9–100)	98.1 (95.1–99.4)	98.2 (95.4–99.5)
Positive (titer ≥ 10)	14	4			
Negative (titer < 10)	0	209			
DENV			100 (83.1–100)	91.2 (86.4–94.4)	92.1 (87.8–95.0)
Positive (titer ≥ 10)	23	18			
Negative (titer < 10)	0	186			

### Neutralization titers.

The primary-screen-positive serum specimens were subjected to confirmatory testing by determination of their RVNT_90_ values. As shown in Table 2, the RVNT primary screen identified 18 ZIKV RVNT-positive samples and 41 DENV-RVNT-positive specimens; notably, all 18 ZIKV RVNT-positive samples were positive in the DENV RVNT. Besides these 41 primary-screen-positive samples, we tested 31 additional ZIKV- and/or DENV-positive serum specimens (defined by known PRNT 90% neutralizing titer [PRNT_90_] values of ≥10) from the New York State Department of Health (NYSDOH) with the confirmatory RVNT. The neutralization antibody titers derived from the RVNT and PRNT methods were compared for each of the 72 serum specimens (Table 3), among which 67 specimens (93.1%) showed equivalent neutralization titers with the ZIKV RVNT and PRNT (differences of ≤4-fold) and 55 samples (76.4%) displayed equivalent neutralization titers with the DENV RVNT and PRNT (Table 4).

**TABLE 3:** Neutralization titers from the RVNT and the PRNT for both ZIKV and DENV (*n* = 72)

Specimen	RVNT screening result^*a*^		RVNT_90_^*b*^		Evidence of infection from RVNT_90_ results	PRNT_90_^*b*^		Evidence of infection from PRNT_90_ results
	ZIKV	DENV	ZIKV	DENV		ZIKV	DENV	
UTMB-1 to -10^*c*^	−	+	<10	10	DENV	<10	<10	No flavivirus
UTMB-11 to -13^*c*^	−	+	<10	20	DENV	<10	<10	No flavivirus
UTMB-14 to -16^*c*^	−	+	<10	40	DENV	<10	10	DENV
UTMB-17	−	+	<10	*80*	DENV	<10	<*10*	No flavivirus
UTMB-18 and -19^*c*^	−	+	<10	*80*	DENV	<10	*10*	DENV
UTMB-20	−	+	<10	*160*	DENV	<10	*10*	DENV
UTMB-21	−	+	<10	*160*	DENV	<10	*20*	DENV
UTMB-22	−	+	<10	160	DENV	<10	40	DENV
UTMB-23	−	+	<10	*320*	DENV	<10	*40*	DENV
UTMB-24	+	+	10	20	Flavivirus	<10	<10	No flavivirus
UTMB-25	+	+	10	40	Flavivirus	<10	10	DENV
UTMB-26	+	+	10	*80*	Flavivirus	<10	*10*	DENV
UTMB-27	+	+	10	160	Flavivirus	<10	40	DENV
UTMB-28	+	+	40	*320*	Flavivirus	10	*20*	Flavivirus
UTMB-29	+	+	40	*640*	Flavivirus	10	*40*	Flavivirus
UTMB-30	+	+	**80**	*320*	Flavivirus	**10**	*20*	Flavivirus
UTMB-31	+	+	160	640	Flavivirus	160	160	Flavivirus
UTMB-32	+	+	**160**	*1,280*	Flavivirus	**10**	*80*	Flavivirus
UTMB-33	+	+	320	*80*	Flavivirus	160	*10*	Flavivirus
UTMB-34	+	+	**320**	*640*	Flavivirus	**10**	*80*	Flavivirus
UTMB-35	+	+	**320**	*1,280*	Flavivirus	**20**	*80*	Flavivirus
UTMB-36	+	+	640	10	Flavivirus	1,280	<10	ZIKV
UTMB-37	+	+	**640**	*5,120*	Flavivirus	**40**	*160*	Flavivirus
UTMB-38	+	+	1,280	20	Flavivirus	2,560	<10	ZIKV
UTMB-39	+	+	1,280	*320*	Flavivirus	2,560	*40*	Flavivirus
UTMB-40	+	+	5,120	*40*	Flavivirus	2,560	<*10*	ZIKV
UTMB-41	+	+	5,120	2,560	Flavivirus	2,560	2,560	Flavivirus
NYSDOH-1	ND	ND	<10	80	DENV	<10	40	DENV
NYSDOH-2	ND	ND	40	320	Flavivirus	10	80	Flavivirus
NYSDOH-3	ND	ND	80	<10	ZIKV	40	<10	ZIKV
NYSDOH-4	ND	ND	160	<10	ZIKV	80	<10	ZIKV
NYSDOH-5	ND	ND	160	<10	ZIKV	320	<10	ZIKV
NYSDOH-6	ND	ND	320	<10	ZIKV	640	<10	ZIKV
NYSDOH-7	ND	ND	320	640	Flavivirus	80	320	Flavivirus
NYSDOH-8	ND	ND	320	640	Flavivirus	160	640	Flavivirus
NYSDOH-9	ND	ND	320	1,280	Flavivirus	160	2,560	Flavivirus
NYSDOH-10	ND	ND	640	<10	ZIKV	160	<10	ZIKV
NYSDOH-11	ND	ND	640	<10	ZIKV	320	<10	ZIKV
NYSDOH-12	ND	ND	640	<10	ZIKV	640	<10	ZIKV
NYSDOH-13	ND	ND	640	<10	ZIKV	1,280	<10	ZIKV
NYSDOH-14	ND	ND	640	20	Flavivirus	320	<10	ZIKV
NYSDOH-15	ND	ND	640	320	Flavivirus	320	160	Flavivirus
NYSDOH-16 to -17^*c*^	ND	ND	1,280	1,280	Flavivirus	320	2,560	Flavivirus
NYSDOH-18	ND	ND	1,280	<10	ZIKV	640	<10	ZIKV
NYSDOH-19	ND	ND	1,280	<10	ZIKV	1,280	<10	ZIKV
NYSDOH-20	ND	ND	1,280	320	Flavivirus	2,560	320	Flavivirus
NYSDOH-21	ND	ND	1,280	1,280	Flavivirus	1,280	1,280	Flavivirus
NYSDOH-22	ND	ND	1,280	5,120	Flavivirus	1,280	20,480	Flavivirus
NYSDOH-23	ND	ND	2,560	1,280	Flavivirus	640	640	Flavivirus
NYSDOH-24 to -25^*c*^	ND	ND	2,560	1,280	Flavivirus	1,280	1,280	Flavivirus
NYSDOH-26	ND	ND	2,560	1,280	Flavivirus	1,280	5,120	Flavivirus
NYSDOH-27	ND	ND	2,560	1,280	Flavivirus	2,560	1,280	Flavivirus
NYSDOH-28	ND	ND	2,560	1,280	Flavivirus	2,560	2,560	Flavivirus
NYSDOH-29	ND	ND	2,560	2,560	Flavivirus	640	1,280	Flavivirus
NYSDOH-30	ND	ND	2,560	2,560	Flavivirus	640	2,560	Flavivirus
NYSDOH-31	ND	ND	2,560	2,560	Flavivirus	5,120	2,560	Flavivirus

a −, negative; +, positive; ND, not done due to limited sample amounts.

b Italic type indicates samples that had >4-fold differences in titers between the DENV RVNT_90_ and DENV PRNT_90_ results. Bold type indicates samples that had >4-fold differences in titers between the ZIKV RVNT_90_ and ZIKV PRNT_90_ results.

c The results for these specimens were identical and therefore were combined in one row.

**TABLE 4:** Neutralization antibody titer agreement between RVNT_90_ and PRNT_90_ results (*n* = 72)

Virus tested	No. (%) of samples with agreement^*a*^	No. (%) of samples with disagreement^*b*^
ZIKV	67 (93.1)	5 (6.9)
DENV	55 (76.4)	17 (23.6)

a Titers derived from RVNT_90_ and PRNT_90_ results showed a ≤4-fold difference; percentages indicate the number of samples with agreement/total number of samples × 100.

b Titers from RVNT_90_ and PRNT_90_ results showed a >4-fold difference; percentages indicate the number of samples with disagreement/total number of samples × 100.

### Interpretation of neutralization antibody titers.

Based on the CDC interpretation guidelines for PRNT diagnosis, samples with PRNT_90_ values of ≥10 for ZIKV and <10 for DENV are interpreted as ZIKV infection, samples with PRNT_90_ values of <10 for ZIKV and ≥10 for DENV are interpreted as DENV infection, and samples with PRNT_90_ values of ≥10 for ZIKV and ≥10 for DENV are interpreted as flavivirus infection (Table 5). When these guidelines were applied to our RVNT confirmatory results, the RVNT detected 10 ZIKV, 24 DENV, and 38 flavivirus infections, while the PRNT identified 14 ZIKV, 13 DENV, and 30 flavivirus infections (Table 5).

**TABLE 5:** Interpretation of neutralization antibody titers

Titer		Interpretation	No. of cases determined by:	
ZIKV	DENV		PRNT	RVNT
≥10	<10	Evidence of ZIKV infection; timing cannot be determined	14	10
<10	≥10	Evidence of DENV infection; timing cannot be determined	13	24
≥10	≥10	Evidence of flavivirus infection; specific virus and timing cannot be determined	30	38
<10	<10	No flavivirus infection	15	0

### Analytical specificity.

The analytical specificity of the RVNT was evaluated with patient serum samples with potentially cross-reactive antibodies and interfering substances. Two groups of specimens were included for interference testing (Table 6). Group 1 included 170 clinical serum samples from patients with flavivirus IgM/IgG, nonflavivirus infections, antinuclear antibodies, anti-mouse antibodies, or elevated rheumatoid factor, cholesterol, bilirubin, or albumin levels. Group 2 consisted of 45 samples spiked with albumin, bilirubin, cholesterol, or hemoglobin. As summarized in Table 6, the presence of anti-DENV antibodies caused cross-reactivity in the ZIKV RVNT for 4 (20%) of 20 samples. Six (50%) of 12 specimens spiked with a high concentration of hemoglobin (90 mg/ml) showed interference in the RVNT, whereas a low concentration (2 mg/ml) did not show any interference. No other components interfered with the RVNT.

**TABLE 6:** Cross-reactivity of potentially interfering substances

Disease/infectious agent or substance	No.		
	Total samples	Expected ZIKV RVNT-negative result	False-positive result with RVNT
Flaviviruses			
DENV	20	16	4
WNV	10	10	0
Yellow fever virus, postimmunization	5	5	0
Other viruses/diseases			
Chikungunya virus	5	5	0
Cytomegalovirus	7	7	0
Epstein-Barr virus, capsid antigen or nuclear antigen	10	10	0
Parvovirus B19	4	4	0
Varicella-zoster virus	16	16	0
Hepatitis C virus	5	5	0
Hepatitis A virus	6	6	0
Hepatitis B virus, surface antigen	14	14	0
Herpes simplex virus 1/2	8	8	0
Rubella virus	11	11	0
Measles virus	5	5	0
Mumps virus	4	4	0
Syphilis	4	4	0
Toxoplasma	1	1	0
Antinuclear antibodies	12	12	0
Elevated rheumatoid factor level (>100 IU/ml)	11	11	0
Potential interfering substances			
Human anti-mouse antibody (>500 ng/ml)	3	3	0
Elevated cholesterol level (>270 mg/dl)	3	3	0
Elevated bilirubin level (>10 mg/dl)	3	3	0
Albumin (4.5 g/dl)	3	3	0
Potential interfering substances (spiked)			
Albumin (60 mg/ml)	7	7	0
Bilirubin (0.4 mg/ml)	7	7	0
Cholesterol (5 mg/ml)	7	7	0
Hemoglobin (2 mg/ml)	12	12	0
Hemoglobin (90 mg/ml)	12	6	6

### Reproducibility.

For assessment of the reproducibility of the RVNT, 65 serum samples (30 negative and 35 positive) from the original screening assay were blindly tested by a second operator on a different day. The reproducibility of the 1:10 dilution screening assay was 100%. For the 35 positive samples, the neutralization titers determined by the two different operators were within a 2-fold range.

## DISCUSSION

A definitive ZIKV diagnosis is made by detecting ZIKV RNA, but the detection period is limited to the first 14 days following ZIKV exposure or symptom onset. For individuals outside this viremia time frame, serological testing with a MAC-ELISA is recommended, according to the diagnostic guidelines from the CDC. A presumptive positive or equivocal MAC-ELISA result needs to be verified with a confirmatory PRNT. Using the current algorithm, the turnaround time to a final result can be weeks or longer. Here we validated a method in which a primary screen followed by a secondary confirmation (to determine the neutralizing antibody titer) can be completed in 4 days using the RVNT. Since the primary screen takes less than 48 h, the diagnostic results of negative for infection could be released in less than 2 days. For samples with positive primary screen results, the secondary confirmation takes another 24 h and the final diagnostic results could be reported within 4 days.

Since PRNT remains the gold standard for a flavivirus serological test, we used the PRNT results as the reference to validate the new RVNT. We evaluated the performance of RVNT as a screen (*n* = 227) and a confirmatory test (*n* = 72). Compared with the PRNT results, the RVNT screens for ZIKV and DENV displayed excellent diagnostic accuracy of 98.2% and 92.1%, sensitivity of 100% and 100%, and specificity of 98.1% and 91.2%, respectively. Compared with the PRNT results, the RVNT as a screen produced 18 discrepant results, including 4 specimens that were both ZIKV and DENV positive and 14 specimens that were ZIKV negative but DENV positive (Table 2). The 4 ZIKV- and DENV-positive specimens produced ZIKV RVNT_90_ values of 10 and DENV values ranging from 20 to 160, indicating a high probability of DENV infection and a low probability of ZIKV infection (i.e., the low ZIKV RVNT titer may be derived from the cross-reactivity of DENV-reactive antibodies). Among the 14 ZIKV-negative but DENV-positive specimens, only the DENV RVNT_90_ produced neutralizing antibody titers (range, 10 to 80), suggesting that a ZIKV infection can be ruled out in specimens that produce only DENV-positive screen results. Whether there is true ZIKV infection in specimens that show both ZIKV- and DENV-positive primary RVNT screen results could be indicated by the relative titers for the two viruses during the second RVNT confirmatory test. For example, specimen UTMB-38 exhibited ZIKV and DENV RVNT_90_ values of 1,280 and 20, respectively (Table 3); it is likely that the DENV titer of 20 was due to the cross-neutralizing activity of the ZIKV-reactive antibodies.

As a confirmatory method, the titers from the RVNT and the PRNT displayed 93.1% and 76.4% agreement for ZIKV and DENV, respectively. The decrease in performance characteristics of the DENV confirmatory test is likely due to greater sensitivity of the RVNT than the PRNT. This observation is in agreement with studies reporting that the neutralization titers measured by a single-round infection assay using WNV-green fluorescent protein (GFP) replicon particles were higher than the traditional plaque assay results (10) and the neutralization titers derived from the reporter ZIKV and DENV assays were on average 2.4- to 2.5-fold higher than those derived from the corresponding ZIKV and DENV plaque assays (9). The greater sensitivity of the RVNT led to more samples being diagnosed with RVNT_90_ values of ≥10. Following the current CDC guidelines for PRNT diagnosis (Table 5), the RVNT results tended to shift the diagnostic conclusion toward positive. One possible solution to decrease the discrepancy between the RVNT and PRNT results would be to increase the cutoff value for the RVNT to a titer that is equivalent to the PRNT titer of 10. For such normalization to be accurate, however, we need more patient sample numbers than those reported in this study. Importantly, no possible ZIKV and DENV infections were missed by the current confirmatory RVNT method.

The analytical specificity of the RVNT was evaluated by testing serum specimens with IgM or IgG against various microorganisms, autoantibodies, and other disease states. Similar to other ZIKV serological assays, cross-reactivity of 20% with the ZIKV RVNT was seen in specimens with IgM/IgG antibodies against DENV (11, 12). We also found that a high concentration of hemoglobin in serum (90 mg/ml) interfered with the ZIKV RVNT but slightly hemolyzed serum (2 mg/ml hemoglobin in serum) did not. Therefore, grossly bloody serum specimens should not be accepted for ZIKV screening and confirmatory testing.

There were a few limitations in this study. First, the DENV neutralization titers were determined only against DENV-2. While the addition of DENV-1 can aid in determining the source of cross-reactivity, the performance of the RVNT as a diagnostic ZIKV assay remains the same. Second, uncharacterized clinical specimens were used to compare the RVNT and the PRNT. Although the RVNT performed well against the PRNT, fully characterized ZIKV and DENV serum specimens from recent and past infections need to be tested to determine the true performance characteristics of the RVNT. However, it is challenging to obtain such well-characterized patient specimens with sequential dual infections. Additionally, a comparison between the RVNT, MAC-ELISA, and PRNT is warranted, to determine the optimal algorithm for identifying ZIKV infections.

Overall, the RVNT displayed excellent performance characteristics as a ZIKV screen and confirmatory test, compared to the PRNT method. The high-throughput capability and rapid turnaround time make the RVNT method useful for the serological detection of ZIKV infections in clinical specimens. Testing more well-characterized patient samples will further strengthen the validation of the new RVNT.

## MATERIALS AND METHODS

### Serum specimens.

A total of 227 clinical serum specimens were collected from low- and high-risk patients. The low-risk group (*n* = 125) contained residual serum specimens from individuals who had not traveled to ZIKV-affected areas after 2013, which were submitted for non-ZIKV-related testing. The high-risk group (*n* = 102) contained serum specimens collected ≥14 days after potential ZIKV exposure or symptoms, from patients who met the CDC clinical and epidemiological testing criteria (which include symptoms, pregnancy, and exposure risk) (8). An additional 31 serum specimens with positive ZIKV and/or DENV PRNT results were provided by the NYSDOH. All of the serum specimens were heated at 56°C for 30 min prior to testing. All diagnostic experiments were performed in a biosafety level 2 laboratory.

### Cells and viruses.

Vero cells (ATCC, Bethesda, MD) were maintained at 37°C with 5% CO_2_ in high-glucose Dulbecco's modified Eagle's medium (DMEM) supplemented with 10% fetal bovine serum (FBS) (HyClone Laboratories, South Logan, UT) and 1% penicillin-streptomycin. The traditional PRNT was performed using the ZIKV Puerto Rico strain PRVABC59 and the DENV-2 New Guinea (NGC) strain. Renilla luciferase ZIKV (strain FSS13025) and DENV-2 (strain NGC) were prepared from previously constructed infectious cDNA clones (13, 14).

### Reporter virus neutralization test.

The assay was performed according to the method described by Shan et al. (9). In brief, Vero cells (1.5 × 10^4^ cells in 50 μl medium, without phenol red, per well) were seeded in a white opaque 96-well plate (Corning). After overnight incubation, the cells were infected with reporter ZIKV or DENV that had been preincubated with patient serum at 37°C for 60 min. At 24 h postinfection, luciferase substrate was added to the infected cells and quantified with a Cytation 5 cell imaging multimode reader (BioTek). Screening samples were diluted 1:10, and samples with ≥90% reduction in luciferase activity were considered reactive. All reactive samples were referred for confirmatory neutralization antibody titer testing, where patient serum was serially diluted from 1:10 to 1:5,120 to determine the RVNT_90_. The 90% neutralization titers were interpreted according to the method described by Rabe et al. (15).

### Plaque reduction neutralization test.

A standard plaque assay was performed with each patient specimen according to the method described by Shan et al. (13). In brief, Vero cells were seeded to 12-well plates (Corning) 1 day before viral infection. On day 2, patient serum was serially diluted and mixed with 100 PFU of either ZIKV Puerto Rico strain PRVABC59 or DENV-2 New Guinea strain. The mixtures were incubated at 37°C for 1 h and inoculated onto confluent Vero cells in 12-well plates. During the 1-h incubation period, the plates were swirled every 15 min to ensure complete coverage of the cell monolayer, for even infection. After the 1-h incubation, 1 ml of methyl cellulose overlay containing 2% FBS and 1% penicillin-streptomycin was added to each well, and the plates were incubated at 37°C for 4 days. Following the incubation, the plates were fixed with 3.7% formaldehyde and incubated at room temperature for 20 min. After removal of the fixative, the plates were stained with 1% crystal violet for 1 min. Visible plaques were counted, and the results were used to determine the PRNT_90_. The 90% neutralization titers were interpreted according to the method described by Rabe et al. (15).

### Analytical specificity.

The potential for cross-reactivity or interference was evaluated by screening ZIKV-negative patient serum samples with the confirmed presence of IgM/IgG antibodies to various microorganisms, autoantibodies, or other disease states. A total of 170 samples were tested, including 35 flavivirus IgM/Ig-positive specimens, 100 IgM/IgG-positive specimens from patients with nonflavivirus diseases, and 35 specimens containing antinuclear antibodies, anti-mouse antibodies, or elevated rheumatoid factor, cholesterol, bilirubin, or albumin levels. Serum samples with ≥90% reduction in luciferase activity were considered cross-reactive or interfering with the ZIKV RVNT. Additionally, both ZIKV-positive and ZIKV-negative samples were spiked with potentially interfering substances that can be found in patient serum, including albumin (60 mg/ml), bilirubin (0.4 mg/ml), cholesterol (5 mg/ml), and hemoglobin (2 mg/ml and 90 mg/ml). The ZIKV RVNT_90_ for each spiked sample was compared to the initial ZIKV RVNT_90_ to determine cross-reactivity or interference.

### Reproducibility.

Thirty ZIKV-negative samples and 35 ZIKV-positive samples with different titers were tested with the RVNT by a second operator on a different day.

### Ethics statement.

This study was conducted under a research protocol approved by the University of Texas Medical Branch (UTMB) institutional review board (protocol 08-182).
